# Peripheral B-Cell Immunophenotyping Identifies Heterogeneity in IgG4-Related Disease

**DOI:** 10.3389/fimmu.2021.747076

**Published:** 2021-09-17

**Authors:** Jieqiong Li, Zheng Liu, Panpan Zhang, Wei Lin, Hui Lu, Yu Peng, Linyi Peng, Jiaxin Zhou, Mu Wang, Hua Chen, Lidan Zhao, Li Wang, Chenman Qin, Chaojun Hu, Xiaofeng Zeng, Yan Zhao, Yunyun Fei, Wen Zhang

**Affiliations:** ^1^Department of Rheumatology, National Clinical Research Center for Dermatologic and Immunologic Diseases, State Key Laboratory of Complex Severe and Rare Diseases, Peking Union Medical College Hospital, Chinese Academy of Medical Science and Peking Union Medical College, Beijing, China; ^2^Department of Rheumatology and Immunology, The First Affiliated Hospital of Zhengzhou University, Zhengzhou, China; ^3^Department of Rheumatology, Hebei General Hospital, Shijiazhuang, China; ^4^Department of Stomatology, Peking Union Medical College Hospital, Chinese Academy of Medical Science and Peking Union Medical College, Beijing, China; ^5^Department of Rheumatology and Immunology, People’s Hospital of Jiaozuo City, Jiaozuo, China

**Keywords:** B-cell subsets, immunophenotyping, cluster analysis, heterogeneity, IgG4-RD

## Abstract

**Objectives:**

To elucidate heterogeneity of IgG4-related disease (IgG4-RD) based on B cell immunophenotyping.

**Methods:**

Immunophenotyping of 4 B-cell subsets in peripheral blood from patients with active IgG4-RD (aIgG4-RD, n=105) was performed using flow cytometry to get preliminary B-cell heterogeneity spectrum. Then 10 B-cell subsets were characterized in aIgG4-RD (n = 49), remissive IgG4-RD (rIgG4-RD, n = 49), and healthy controls (HCs, n = 47), followed by principal components analysis (PCA) and cluster analysis to distinguish B-cell immunophenotypes and classify IgG4-RD patients into subgroups.

**Results:**

Cluster analysis identified two endotypes in 105 aIgG4-RD patients based on 4 B-cell subsets: Group1 with higher Breg and naive B cells (n = 48), and Group2 with higher plasmablasts and memory B cells (MBCs) (n = 57). PCA indicated that aIgG4-RD consisted of plasmablast-naive B cell and MBCs-Breg axes abnormalities. There was a negative relationship between naive B cells and disease activity. Both plasmablasts and MBCs were positively associated with serological biomarkers. Cluster analysis stratified aIgG4-RD patients into 3 subgroups based on 10 B-cell subsets: subgroup1 with low MBCs and normal Breg, subgroup2 with high MBCs and low Breg, and subgroup3 with high plasmablasts and low naive B cells. Patients in subroup2 and subgroup3 were more likely to be resistant to treatment.

**Conclusion:**

Patients with aIgG4-RD can be divided into 3 subgroups based on B cell heterogeneity. The B cell immunophenotyping could help elucidate the pathogenesis of IgG4-RD, identify patients with potential refractory IgG4-RD, and provide important information for the development of new therapies.

## Introduction

IgG4-related disease (IgG4-RD) is an immune-mediated fibrotic disease characterized by elevated serum IgG4 concentration, tissue infiltration by IgG4+ plasma cells, and a marked responsiveness to both glucocorticoids and B cell depletion with rituximab (RTX) (1). Various immunologic abnormalities contribute to generating the inflammatory masses in IgG4-RD, including M2 macrophages (2), activated B cells (3), CD4+ CTLs (4) and other immune-related cells.

The pathogenic role of B cell subsets has been given increasing attention since IgG4-RD was first recognized. In particular, plasmablasts are highly expanded and infiltrate tissue with extensive somatic hypermutation (5). Circulating plasmablasts are a useful biomarker, and correlate with other clinical and serological biomarkers of IgG4-RD activity (6, 7). B cell depletion with RTX has been proved effective for the treatment of IgG4-RD, which validates the suggested pathogenicity of B cells in this disease (8). Glucocorticoids (GCs), on the other hand, are not supposed to affect the total number of circulating B cells in IgG4-RD, but reduce naïve B cell, increase memory B cells (MBCs), and deplete circulating plasmablasts (9). The increase of circulating memory B cells after 6 months of GCs treatment might predict IgG4-RD relapse (10).

Taken together, the above findings suggest B-cell compartment in IgG4-RD is phenotypically heterogeneous. Although RTX is effective for both induction therapy and treatment of relapses in IgG4-RD, the high rate of infections and the temporary effect of RTX might be hindrances to such strategy (11), for it depletes B cell crudely, and B cell reconstitution is inevitable. Previous study has identified four homogenous clinical phenotypes based on typical patterns of organ involvement (12): Pancreato-Hepato-Biliary disease, Retroperitoneal Fibrosis and/or Aortitis, Head and Neck-Limited disease, and classic Mikulicz syndrome with systemic involvement. But patterns of B-cell subsets remain poorly defined. Moreover, the full characterization of circulating B-cell subsets in IgG4-RD patients at different stages of disease activity compared with healthy controls (HCs) was not carried out, which could offer a better understanding of their involvement in the pathogenesis of IgG4-RD.

Based on these considerations, it is worth raising three clinical questions: 1) What are the differences in B-cell immunophenotypes between IgG4-RD patients and healthy individuals, before and after treatment? 2) How do the B-cell immunophenotypes interact? 3) Can patients be divided into subgroups by immunophenotyping? 4) What’s the association of B-cell subsets abnormalities and clinical phenotypes (12)? To address these questions, we initially characterized 10 B-cell subsets in IgG4-RD, and tried to obtain a broader perspective on the B-cell heterogeneity in IgG4-RD by immunophenotyping.

## Methods

### Study Subjects

Peripheral blood (PB) was obtained from patients with active IgG4-RD (aIgG4-RD, n=105), remissive IgG4-RD (rIgG4-RD, n=49), and HC (n=47). IgG4-RD was diagnosed according to the 2011 comprehensive IgG4-RD diagnostic criteria (13) and the 2019 American College of Rheumatology/European League Against Rheumatism classification criteria for IgG4-RD (14). Patients with infectious diseases, other rheumatic diseases, malignancies, or conditions that could mimic IgG4-RD were excluded. HCs were matched for gender and age. All subjects were enrolled in accordance with ethics regulations, approved by the Ethics Committee of Peking Union Medical College Hospital, following written informed consent.

### Laboratory Analysis and Flow Cytometry

Laboratory analyses of IgG4-RD patients before and after treatments included percentage of eosinophil (EOS%), absolute eosinophil count (AEC), C-reactive protein (CRP), erythrocyte sedimentation rate (ESR), complement (C3 and C4), immunoglobin (IgG, IgA, IgM, and T-IgE), IgG1, IgG2, IgG3, and IgG4 subclasses. Peripheral blood mononuclear cells (PBMCs) were isolated from PB using Ficoll-Hypaque density gradient centrifugation. 1X10^6^ PBMCs were stained for B-cell subsets for 30 minutes at 4°C after washing and re-suspend in cell staining buffer. Flow cytometric analysis was performed immediately after sample preparation (see **Supplementary Figure 1** for gating strategy). All samples were analyzed using a BD FACSAria II system (BD Biosciences), and data were analyzed using FlowJo software (Tree Star).

### Treatments and Clinical Assessment

The treatments for IgG4-RD patients were classified into four categories: watchful waiting, GCs monotherapy, immunosuppressive agents (IM) monotherapy, and GCs + IM combination. IM applied in our study was graded as strong potency IM including cyclophosphamide (CTX) and mycophenolate mofetil (MMF), and weak potency IM including methotrexate (MTX) and leflunomide (LEF). The average follow-up period was 20 months. Remissive IgG4-RD included complete remission (CR) and partial remission (PR): CR was defined as IgG4-RD RI (2018) =0; PR was defined as IgG4-RD RI (2018) declining by ≥ 50%. Relapse was defined as a recurrence of symptoms and signs and/or worsening of imaging studies, with or without re-elevation of the serum IgG4 level. Potential refractory IgG4-RD was defined as no significant improvement on serological biomarkers especially reported risk factors (15, 16), although remission was achieved.

### Study Design

We preliminarily evaluated the differences in B-cell immunophenotype in 105 aIgG4-RD patients. PBMCs were stained with CD19, CD24, and CD38 antibodies to obtain 4 B-cell subsets (CD19^+^CD24^+^CD38^−^ MBCs, CD19^+^CD24^int^CD38^int^ naïve B cells, CD19^+^CD24^hi^CD38^hi^ regulatory B cells (Bregs), and CD19^+^CD24^−^CD38^hi^ plasmablasts cells) (17), which was followed by cluster analyses to get a preliminary B-cell heterogeneity spectrum.

The gating strategy of B-cell subpopulations varies under the scientific research focus. Therefore, we further investigated and compared 10 published B-cell subsets (17–19) in aIgG4-RD (n=49), rIgG4-RD (n=49), and HCs (n=47), including 3 plasmablasts cells (CD19^+^CD24^-^CD38^hi^, CD19^+^CD27^hi^CD38^hi^, and CD19^+^IgD^-^CD38^hi^), 2 naive B cells (CD19^+^CD24^int^CD38^int^, CD19^+^IgD^+^CD38^±^), 4 MBCs (CD19^+^IgD^-^CD27^+^, CD19^+^CD24^+^CD38^-^, CD19^+^IgD^+^CD27^+^, and CD19^+^IgD^-^CD38^-^CD27^+^), and CD19^+^CD24^hi^CD38^hi^ Breg (**Supplementary Figure 1**). Principal components analysis (PCA) was performed to reduce the dimensionality of immunophenotyping data and followed by cluster analysis to classify the mixed samples (patients and HCs) into subgroups.

To elucidate the diversity among patients with aIgG4-RD, we performed a separate analysis of 49 aIgG4-RD using PCA and cluster analysis again. Disease activity associated changes after treatment were analyzed when these patients obtained clinical remission after treatment, including EOS%, ESR, IgG4, and T-IgE.

**Figure 1:**
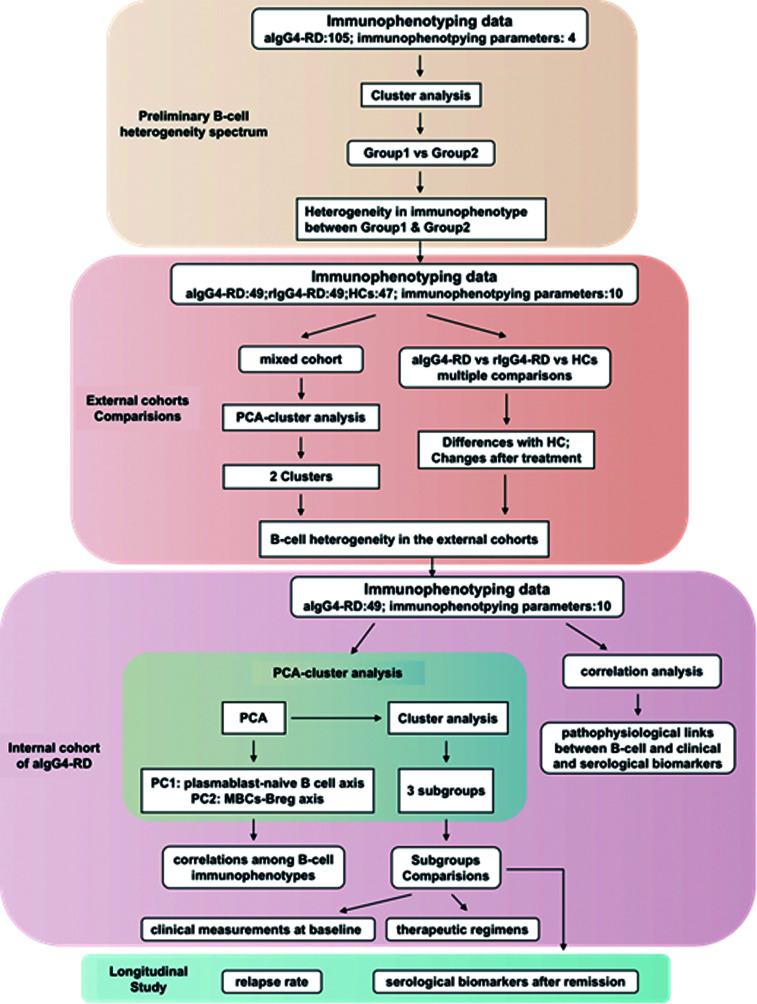
The flow scheme of study design. HCs, (healthy controls); aIgG4-RD, (active IgG4-RD); rIgG4-RD, (remissive IgG4-RD); PCA, (principal components analysis).

The immunophenotyping data and/or clinical features were compared among the different populations including HCs, aIgG4-RD, rIgG4-RD patients and the classified patients’ groups. **Figure 1** presents information on the study design in flowchart format.

### Statistical Analysis

Statistical analysis was performed using IBM SPSS Statistics version 24.0 software. Data including demographic data, immunophenotyping data, and longitudinal clinical data were stored in Microsoft Excel. Data were reported as mean ± SD. Normal distribution data between two groups were analyzed using independent-samples t tests or paired samples t tests, and one-way analysis of variance (ANOVA) was used among 3 groups. Non-normally distributed data between two groups were compared using the Mann-Whitney U-test, and Kruskall-Wallis was performed among 3 groups. Chi-squared tests were used to compare Categorical variables such as treatment categories, clinical phenotypes, and relapse state. The relationships between B-cell subsets and clinical features were analyzed by Pearson’s or Spearman’s correlation test. Paired t-tests were used to assess differences in serum biomarkers before and after treatments. P-values <0.05 were considered significant.

### Principal Components Analysis and Cluster Analysis

For easy exploration and visualization of multiple variables, we used PCA to statistically aggregate 10 items of B-cell subsets, reducing the dimensionality of immunophenotyping data for subsequent cluster analysis, and exploring the correlation between these variables. According to eigenvalues (λ> 1) and cumulative contribution rate (>75%), we selected appropriate number of eigenvectors with the top highest eigenvalues as principal components (PCs). According to Component Matrix and eigenvalue, PC scores (the values for extracted PCs) were also calculated in individual samples as new variables for further cluster analysis (20, 21).

Cluster analysis was performed three times in this study using a hierarchical and agglomerative clustering algorithm with the Ward method (20–22): directly performed in 105 aIgG4-RD patients with 4 B-cell subsets data, following PCA in a mixed samples with 10 B-cell subsets data, and following PCA in aIgG4-RD patients (n=49) with 10 B-cell subsets data. We determined the number of clusters based on the scree plot (eigenvalue > 1) and tree diagram. In this study, we judged that the appropriate number of clusters was 2 in mixed samples and 3 in 49 aIgG4-RD patients.

## Results

### General B-Cell Subsets Architecture of 105 Active IgG4-RD Patients

Baseline clinical characteristics and 4 B-cell subsets of 105 aIgG4-RD patients were shown in **Supplementary Table 1**. The mean age of diagnosis was 52.5 years, and most patients were males (64.8%). The mean duration of IgG4-RD was 3.1 years; mean diagnostic score (2019 ACR/EULAR classification criteria) and IgG4-RD RI (2018) at baseline were 32.9 and 7.6, respectively.

Cluster analysis classified aIgG4-RD patients into Group1 (n=48) and Group2 (n=57) based on 4 B-cell subsets (**Supplementary Figure 2**). Compared with patients in Group1, patients in Group2 were more likely to be male (75.4%), had shorter disease duration, but more organs involved, higher diagnostic score, higher level of IgG, IgG1, IgG4, T-IgE, and higher IgG4-RD RI. Unsurprisingly, Group2 was characterized by higher plasmablasts and memory B, but lower naive B and Breg (**Supplementary Table 1**). The proportions of B-cell subsets in individual patients in the 2 groups are plotted in three-dimensional diagrams (**Supplementary Figures 3A, B**), which confirmed that aIgG4-RD patients were clearly separated according to these 4 B-cell subsets.

### Cluster Analysis Identified IgG4-RD Patients With High B-Cell Heterogeneity Spectrum Irrespective of Their Disease Activity Compared With Healthy Controls

Based on the observed heterogeneity of B-cell subsets architecture in aIgG4-RD and a variety of B-cell subsets published with different values, we hypothesized that B-cell subsets could be different among aIgG4-RD, rIgG4-RD and HCs. Therefore, we further analyzed 10 B-cell subsets in these three cohorts (aIgG4-RD, n=49; rIgG4-RD, n=49; HCs cohorts, n=47) (**Supplementary Figure 1**), and combined them into one mixed cohort (n=145) for PCA and cluster analysis.

**Figure 2:**
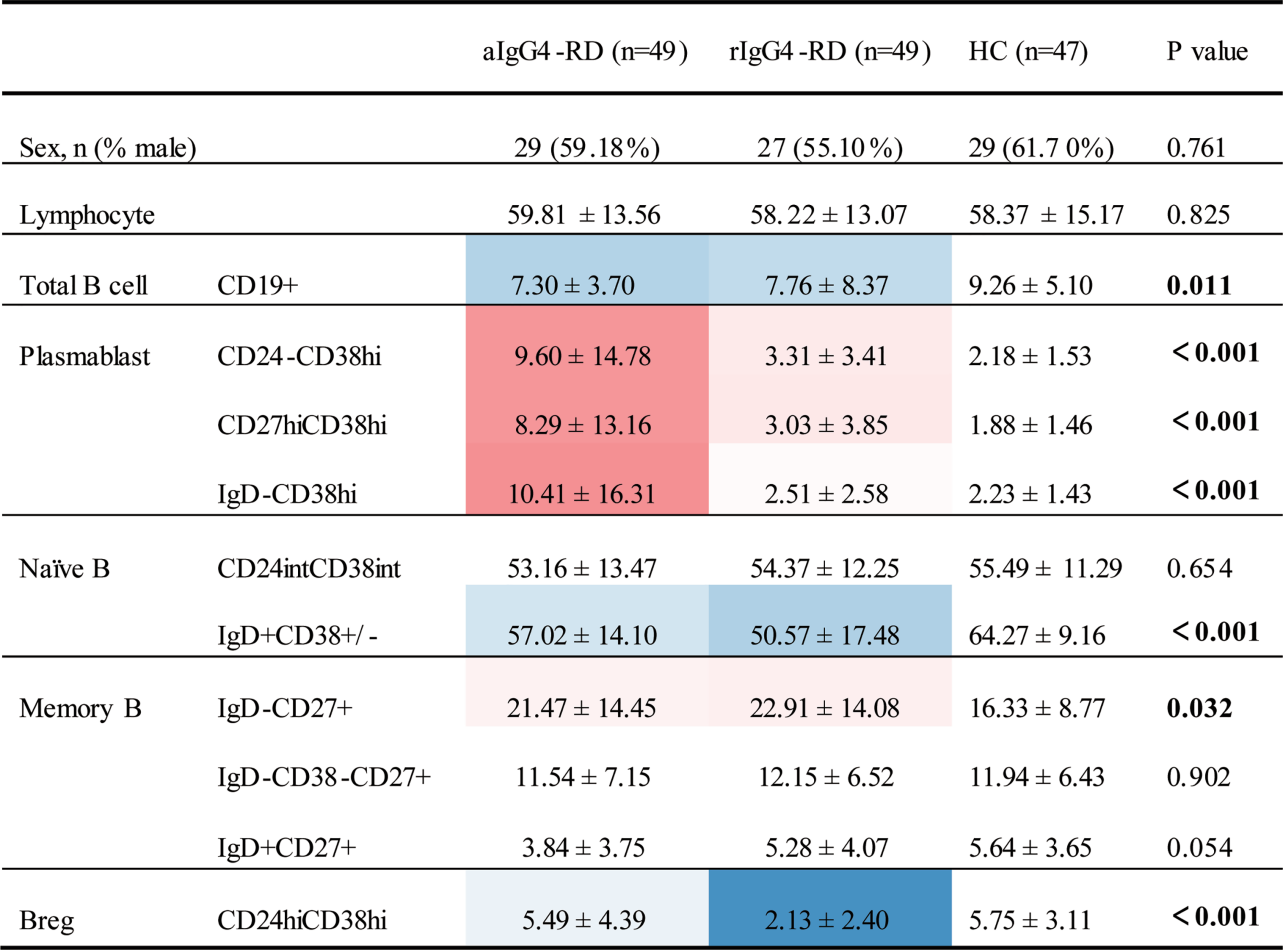
Differences in phenotypes of B- cell subsets among patients with IgG4-RD and age- and sex-matched healthy control subjects. Values that were significantly different in the patient group compared with the healthy control group highlighted in color (blue for decreased; red for increased). aIgG4-RD, active IgG4-RD; rIgG4-RD, remission IgG4-RD.

First, we compared B-cell subsets proportions of the 3 cohorts (**Figure 2** and **Supplementary Table 2**). When compared with HCs, patients with IgG4-RD had lower proportion of CD19^+^ total B cells, lower proportion of CD19^+^IgD^+^CD38^±^ naive B cell, and higher proportion of CD19^+^IgD^-^CD27^+^ MBCs no matter the disease was active or in remission. In addition, there were significant differences in 3 plasmablasts with different markers (CD19^+^CD24^-^CD38^hi^, CD19^+^CD27^hi^CD38^hi^, and CD19^+^IgD^-^CD38^hi^) between aIgG4-RD patients and HCs, which were remarkably higher in aIgG4-RD patients and reduced drastically after treatments in rIgG4-RD patients. The proportion of CD19^+^IgD^+^CD27^+^ unswitched MBCs were lower in aIgG4-RD patients than HCs, and similar to that in rIgG4-RD patients. The result was worth discussing is that CD19^+^CD24^hi^CD38^hi^ Breg proportion did not differ between aIgG4-RD patients and HCs, and decreased after treatments. No significant differences in proportions of CD19^+^CD24^int^CD38^int^ naive B cell and CD19^+^IgD^-^CD38^-^CD27^+^ switched MBCs were observed among cohorts.

We extracted 3 PCs (accumulative contribution rate = 78%) in the mixed cohort based on ten B-cell subsets ratios: PC1 (eigenvalue 4.348), PC2 (eigenvalue 2.395), and PC3 (eigenvalue 1.056). Then two distinct Clusters (based on 10 B-cell subsets profile) were identified by cluster analysis (**Supplementary Figure 4**): Cluster 1 (n=66; aIgG4-RD=32, rIgG4-RD=12, HCs=22) and Cluster 2 (n=79; aIgG4-RD=17, rIgG4-RD=37, HCs=25). Each Cluster included aIgG4-RD, rIgG4-RD and HCs. And there was striking difference in constituent ratio of cohorts between Clusters by chi-square test (P<0.001). Cluster1 more often presented with aIgG4-RD patients than Cluster2 (48.5% vs 21.5%), while Cluster2 more often presented with rIgG4-RD patients than Cluster1 (46.8% vs 18.21%). Healthy controls distributed evenly in two Clusters (33.3% vs 31.7%). As expected, the obvious heterogeneity among individual patients with IgG4-RD and significant differences in B-cell subsets profile before and after treatment were observed visually by heat map (**Supplementary Figure 4**). Together, these results suggest that IgG4-RD patients present with high B-cell heterogeneity spectrum irrespective of their disease activity compared with HCs, especially in aIgG4-RD patients.

### B-Cell Subsets Ratios Correlated With Various Disease-Associated Indexes and Presented Plasmablast-Naive B Cell and MBCs-Breg Axes Abnormalities in aIgG4-RD

To assess a potential pathophysiological link between circulating B-cell subsets frequencies and aIgG4-RD, we performed correlation analysis with the following validated clinical and serological biomarkers in aIgG4-RD cohort (n=49): number of organs involved, IgG4-RD RI (2018), serum IgG, IgG_1-4_ and T-IgE levels, EOS%, AEC, ESR, CRP, and C3 (**Supplementary Table 3**). In general, circulating plasmablasts cells as well as CD19^+^IgD^-^CD27^+^ MBCs ratio showed statistically significant positive correlation with disease activity and disease severity associated indexes, while naive B cells were negatively correlated with these indexes. CD19^+^IgD^+^CD27^+^ unswitched MBCs and CD19^+^CD24^hi^CD38^hi^ Breg proportions only showed negative correlation with T-IgE and ESR, respectively. No statistically significant correlation was found between CD19^+^IgD^-^CD38^-^CD27^+^ switched MBCs and disease-associated indexes. In particular, CRP had no correlation with any B-cell subsets ratio (data not shown).

**Figure 3:**
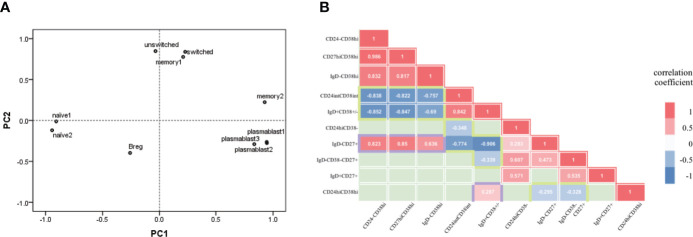
B-cell subsets in active IgG4-RD patients presented plasmablast-naive B- cell and MBCs-Breg axes abnormalities. **(A)** Each B-cell subset immunophenotype was visualized in 2 two dimensions by principal components (PC) analysis. plasmablast1, CD19+CD24-CD38hi; plasmablast2, CD19+CD27hiCD38hi; plasmablast3, CD19+IgD-CD38hi; naïve1, CD19+CD24intCD38int; naïve2, CD19+IgD+CD38±; memory1, CD19+CD24+CD38-; memory2, CD19+IgD-CD27+; switched, CD19+IgD-CD38-CD27+ switched memory B cell; unswitched, CD19+IgD+CD27+ unswitched memory B cell; Breg, CD19+CD24hiCD38hi. **(B)** Correlation between pairs of B-cell types (10 immunological types). Correlation coefficients for each pair of cell types are represented by colour [red=positive correlation coefficient (p<0.05); blue=negative correlation coefficient (p<0.05); green=no significant correlation (p<0.05)].

To further investigate the abnormalities of B-cell subsets in aIgG4-RD, we separately used PCA in aIgG4-RD cohort to statistically aggregate these 10 immunophenotypes. Two PCs (accumulative contribution rate = 77%) were extracted based on ten B-cell subsets ratios: PC1 (eigenvalue 5.214), and PC2 (eigenvalue 2.476) (**Supplementary Figures 5A, B**). The ability of each PC to represent the corresponding B-cell subsets ratio and the correlations among ten immunophenotypes were estimated by correlation coefficient and visualized by a 2-dimensional loading plot, respectively (**Supplementary Figure 5B**, **Figure 3A**). The results showed PC1 was associated with plasmablasts cell and naive B cell phenotype. The positive side of PC1 contained 3 differently labelled plasmablasts cells (CD19^+^CD24^-^CD38^hi^, CD19^+^CD27^hi^CD38^hi^, and CD19^+^IgD^-^CD38^hi^), while the negative side contained 2 types of naive B cells (CD19^+^CD24^int^CD38^int^, CD19^+^IgD^+^CD38^±^). In contrast, PC2 was mainly associated with MBCs and Breg. The positive side of PC2 contained CD19^+^CD24^+^CD38^-^, CD19^+^IgD^-^CD38^-^CD27^+^ switched, and CD19^+^IgD^+^CD27^+^ unswitched MBCs, while the negative side contained CD19^+^CD24^hi^CD38^hi^ Breg. Specially, in memory B phenotype, CD19^+^IgD^-^CD27^+^ MBCs seemed to play a more significant role in PC1 than in PC2.

In addition, we explored the correlations among B-cell immunophenotypes based on the distances among subsets and correlations analysis (**Figures 3A, B**). The loading plot showed clear separation among plasmablasts cells, naive B cell, MBC and Breg. On the whole, plasmablasts cells and naive B cells were located statistically opposite sides in loading plot and showed negative correlations (all pairs, P<0.001). Similarly, MBCs and Breg were locally opposite with negative correlations (CD19^+^IgD^-^CD27^+^ MBCs with Breg, P=0.04; CD19^+^IgD^-^CD38^-^CD27^+^ switched MBCs with Breg, P=0.021). Breg also showed positive correlation with CD19^+^IgD^+^CD38^±^ naive B cell (P=0.046). While both CD19^+^CD24^int^CD38^int^ and CD19^+^IgD^+^CD38^±^ naive B cells were negatively correlated with MBCs, especially CD19^+^IgD^-^CD27^+^ MBCs (P<0.001). Interestingly, CD19^+^IgD^-^CD27^+^ MBCs and plasmablasts cells were statistically close in loading plot and showed great positive correlations (all pairs, P<0.001), both were negatively correlated with naive B cells, and positively correlated with disease activity and severity associated indexes as previously mentioned (**Supplementary Table 3**). The results of correlation analysis and PCA indicated that the immunophenotype of B-cell subsets in aIgG4-RD consists of plasmablast-naive B cell and MBCs-Breg axes abnormalities.

### Cluster Analysis Identified Three Subgroups of Active IgG4-RD Patients

**Figure 4:**
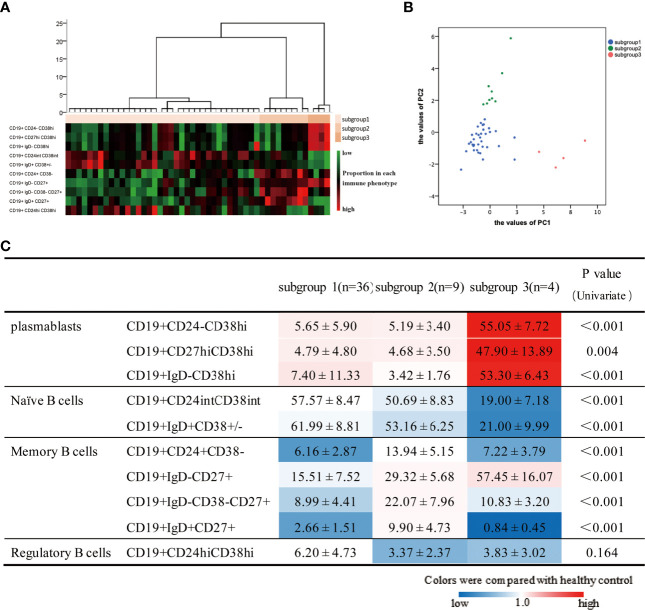
Results of cluster analysis based on 10 B-cell phenotypes in patients with active IgG4-RD. **(A)** Hierarchical statistical clustering of IgG4-RD patients. **(B)** PC1 and PC2 values in individual patients in the 3 three subgroups. **(C)** B-cell subset ratios in 3 three subgroups are shown. Values are the mean ± SD, with levels that were significantly different in the patient subgroup compared with the healthy control group highlighted in color (blue for decreased; red for increased).

Given the heterogeneity and abnormalities of circulating B-cell subsets in patients with IgG4-RD, we next attempted to identify subgroups among 49 aIgG4-RD patients. Cluster analysis revealed that aIgG4-RD patients could be classified into 3 subgroups (subgroup1, n=36; subgroup2, n=9; subgroup3, n=4) (**Figure 4A**). The values of PC1, which was associated with plasmablast-naive B cell, and PC2, which was associated with MBCs-Breg, were calculated in individual samples and plotted in scatter plot (**Figure 4B**), which showed that aIgG4-RD patients were clearly separated and localized into 3 regions according to these 2 axes.

To assess whether the grouping also reflected distinct B-cell immunological profiles, we compared B-cell subset ratios in 3 subgroups as well as between each subgroup and HCs (**Figure 4C**). There were significant differences in all B-cell subsets except for Breg among subgroups. When compared with HCs, higher proportions of plasmablasts cells were observed in all subgroups to varying extents. Subgroup1 had lower MBCs proportions, as well as normal Breg and naive B cells proportions. Subgroup2 had the highest percentages of MBCs overall, but the lowest percentage of Breg. Subgroup3 was characterized by the highest proportions of plasmablasts cells, but the lowest percentage of naive B cells. Furthermore, we also found subgroup3 had the highest proportion of CD19^+^IgD^-^CD27^+^ MBCs but lowest CD19^+^IgD^+^CD27^+^ unswitched MBCs, which suggested there were inseparable relations between CD19^+^IgD^-^CD27^+^ MBCs and plasmablasts again.

### Patients With High MBCs and Plasmablast in Subgroup2&3 Were Potential Treatment-Resistant

To further investigate whether the grouping was clinically meaningful as a potential predictor of disease severity, treatment responses, and outcomes, a wide range of serological biomarker levels were compared among patients in 3 subgroups, as well as clinical measurements were collected longitudinally at subsequent encounters after treatments. Subgroup3 had significantly higher serum IgG, IgG1, IgG3, IgG4, T-IgE levels, and higher EOS%, AEC, ESR, but lower C3 than subgroup1&2 (**Supplementary Table 4**), suggesting patients in subgroup3 had higher disease severity. The number of organs involved and IgG4-RD RI did not differ. And there was no difference in therapeutic regimens (P=0.33), as well as intensity of treatment, including GC initial dose (P=0.681) and IM grades (P=0.513). The mean doses of initial GCs in the 3 subgroups were 41.18mg/d, 43mg/d, and 45mg/d, respectively. In GCs + IM combination therapy, the proportions of strong potency IM applyment were 64.7% in subgroup1, 80% in subgroup2, and 100% in subgroup3. Only three patients experienced relapse, all of them received IM monotherapy and belonged to subgroup1. Recurrence rate (P=0.521) showed no statistic difference among subgroups.

**Figure 5:**
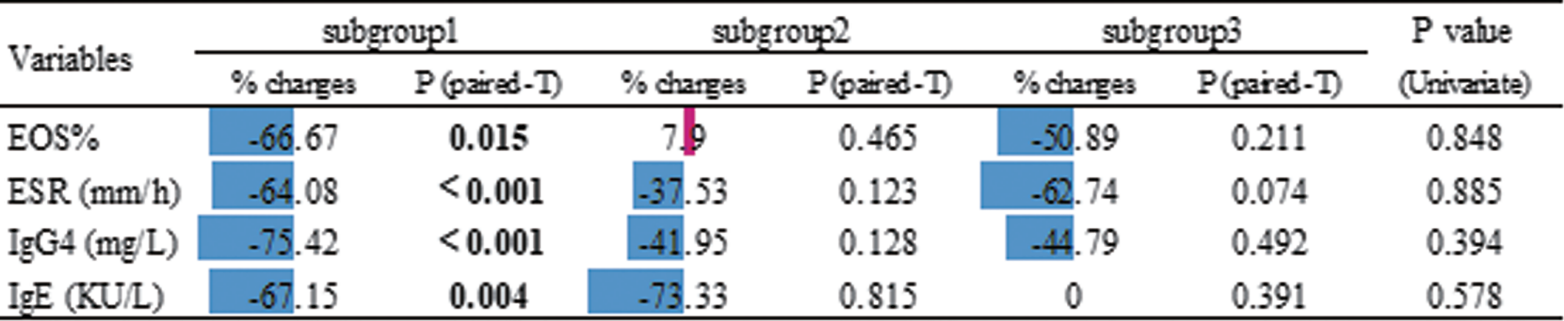
Effects of treatment according to the the changes in serological biomarker levels using Paired paired t-test. The % changes are relative to the baseline. These changes are also shown in the chart using color (blue for decrease and red for increase) and compared among subgroups by univariate analysis (P values shown in the last column).

Next we compared serological biomarker levels after disease achieved remission among 3 subgroups (**Table 1**). Patients in subgroup3 still had the highest levels of EOS%, ESR, IgG4 and T-IgE. Considering subgroup3 had higher disease severity at baseline (**Supplementary Table 4**), we investigated the changes in these biomarker levels using Paired t-tests (**Figure 5**), patients in subgroup1 achieved significantly lower levels of EOS%, ESR, IgG4 and T-IgE than these at baseline, but no statistic difference in subgroup2&3. We also explored the percentages of changes to baseline levels, which didn’t differ among subgroups. These results indicated patients in subgroup2&3 with potential refractory IgG4-RD.

**Table 1:** Serological biomarker levels of IgG4-RD patients in each subgroup after treatment.

Variables	subgroup1	subgroup2	subgroup3	P value (Univariate)
EOS%	1.77 ± 2.08	1.87 ± 1.24	10.00 ± 11.39	**0.001**
ESR (mm/h)	9.52 ± 6.85	18.57 ± 19.07	30.33 ± 27.65	**0.013**
IgG4 (mg/L)	3172.07 ± 3171.98	2359.29 ± 1347.96	22575.00 ± 36497.76	**0.008**
IgE (KU/L)	87.12 ± 94.20	85.10 ± 174.10	788.73 ± 1003.21	**0.002**

P values in the univariate analysis were determined by one-way analysis of variance (ANOVA) or Kruskall-Wallis test. P-values < 0.05 were considered statistically significant. EOS%, percentage of eosinophil; ESR, erythrocyte sedimentation rate; IgG4, immunoglobulin G4.

### Association of B-Cell Subsets Abnormalities and Clinical Phenotypes

We compared the distribution of clinical phenotypes in each group using Chi-squared tests (**Supplementary Figure 6**). Between Group1 and Group2 identified by 4 B-cell subsets, Group1 showed higher proportion of clinical phenotype3, while Group2 showed higher proportion of clinical phenotype4 (P=0.001). Among subgroups identified by 10 B-cell subsets, the majority of patients in these 3 subgroups were those with clinical phenotype3&4, 2&3, and phenotype4, respectively.

We also compared the differences in phenotypes of B cell subsets among patients with different clinical phenotypes. On the whole, all clinical phenotypes had high plasmablasts. Clinical phenotype2 had the highest MBCs, but the lowest Breg. The only abnormality of B-cell subset in clinical phenotype3 was higher plasmablasts, this phenotype had relatively lower MBCs, normal naïve B cells and Breg. Clinical phenotype4 was characterized by the highest plasmablasts, the lowest naive B cells and total B cells, as well as higher MBCs and lower Breg. The features of B-cell subsets in clinical phenotype1 seemed to be intermediate between phenotype3 and phenotype4 (**Supplementary Figure 7**).

## Discussion

Our study represents a new classification of patients with IgG4-RD based on B-cell immunophenotyping, which confirmed the importance of plasmablast-naive B cell and memory B cell-Breg axes in IgG4-RD, established a subgroup of potential refractory IgG4-RD patients with high plasmablasts and memory B cell.

Previous immunophenotyping studies provide several landmark evidences indicating that B cells are central to the pathogenesis of IgG4-RD, including (i) CD19^+^CD24^−^CD38^hi^ plasmablasts cells increase in active IgG4-RD (17); (ii) B cells contribute directly to tissue fibrosis in IgG4-RD, particularly of plasmablasts (3, 23); (iii) plasmablast and CD19^+^IgD^-^CD27^+^CD38^-^ memory B cells decreased after Iguratimod plus GCs treatment (18); (iv) GCs reduce CD19^+^CD20^+^CD27^-^CD38^+^ naive B cell, increase CD19^+^CD20^+^CD27^+^CD38^-^ memory B cells, and deplete plasmablasts (9); (v) increase of circulating CD19^+^IgD^-^CD27^+^CD38^-^ memory B cells after GCs treatment predicts IgG4-RD relapse (10); (vi) RTX is effective for IgG4-RD because it depletes all measurable peripheral B cells (8). We showed for the first time that patients with IgG4-RD have quite a few differences in their B-cell immunological architecture, spanning from the external comparison with healthy controls to the internal comparison among IgG4-RD subgroups.

We sketched out the general picture of B-cell heterogeneity spectrum in aIgG4-RD based on 4 immune cell subsets according to previous reports (17). Despite a predominance of plasmablasts found in IgG4-RD, patients with aIgG4-RD present heterogeneity by clustering: Group2 showed high plasmablast and MBC, low naive B and Breg, as well as a high proportion of male sex, high disease activity and severity; another cluster showed converse characterization. The results suggest clinical and B-cell heterogeneity among IgG4-RD patients as well as a potential pathophysiological link between clinical features and B-cell immunological spectrum. In addition, the results consist with the findings that male sex is associated with high serological markers and worse prognosis (24).

We further explored B-cell heterogeneity in external cohorts including IgG4-RD and HCs, and expanded 4 B-cell subsets into 10 subsets according to previous reports (18, 25, 26). The remarkable difference compared to rIgG4-RD and HCs was higher plasmablasts cells in aIgG4-RD, which has been replicated by our study as well. Compared with HCs, CD19^+^IgD^+^CD38^±^ naive B cells decreased in aIgG4-RD and further declined after treatment. Another study also reported GC-induced disease remission was accompanied by a reduction of CD19^+^CD20^+^CD27^-^CD38^+^ naïve B cells (9). The classical function of MBCs is to retain the capability of rapidly differentiating into plasmablasts in the context of re-exposure to their cognate antigens, while different lymphocyte subsets with opposing functions are now known to be part of the MBCs (27). In our study, CD19^+^IgD^-^CD27^+^ MBCs is increased in aIgG4-RD and no significant change after treatment, while the proportion of CD19^+^IgD^+^CD27^+^ unswitched MBCs were lower in aIgG4-RD patients than HCs. It’s reported that the percentages of CD19^+^IgD^+^CD38^±^ naïve B cells decreased, while CD19^+^IgD^-^CD27^+^ MBCs and plasmablasts increased following stimulation with IgG4-RD plasma exosomes (28). IgD^+^CD27^+^ unswitched MBCs usually present anti-inflammatory properties, for instance, are reduced in systemic lupus erythematosus (26), primary Sjögren’s Syndrome (pSS) (19), and reconstitute after immunosuppressive treatment, this phenomenon also is observed in IgG4-RD in our study. IgG4-RD and pSS share several clinical and serological characteristics, such as enlargement of lacrimal and the salivary glands, and high immunoglobulin level. IgD^+^CD27^+^ unswitched MBCs decrease is an early feature of pSS correlated with serological autoimmunity and disease progression, and represents the loss of a MZ-equivalent endowed with protective functions such as apoptotic clearance, Interleukin-10-mediated B regulatory activity (26). But unswitched MBCs have not previously been studied in IgG4-RD pathogenesis. Our study suggests that CD19^+^IgD^-^CD27^+^ MBCs and IgD^+^CD27^+^ unswitched MBCs may play opposite role in IgG4-RD, the former is pathogenic, the latter is protective. The role of Breg in the context of IgG4-RD is not entirely understood yet. Our data show the proportion of CD19^+^CD24^hi^CD38^hi^ Breg in aIgG4-RD is similar to that in HCs, and decreases after treatment. The previous studies reported CD19^+^CD24^hi^CD38^hi^ Bregs were increased in type 1 AIP patients (29), but another showed IgG4-RD patients had a lower frequency (25). There has been evidences that the profile of patients with different organs lesions differ from that of patients with other type of IgG4-RD. These conflicting results about Breg among studies may be caused by differences in the involved organs of IgG4-RD (30). Cluster analysis identified two clusters within the mixed cohort, which were characterized by differential B-cell immune signatures and had a high discriminatory capacity to identify aIgG4-RD and rIgG4-RD. In addition, both aIgG4-RD-dominant Cluster1 and rIgG4-RD-dominant Cluster2 were composed of a part of HCs. These findings highlight heterogeneous immune-pathogenic features underlying aIgG4-RD and rIgG4-RD. Patients with rIgG4-RD re-experience an exacerbation may causally linked to the abnormal B-cell immunophenotypes.

We further explored high B cell heterogeneity in the internal cohort of aIgG4-RD. PCA and correlation analysis demonstrated relationships among B-cell immunophenotypes in aIgG4-RD. Plasmablasts cells and CD19^+^IgD^-^CD27^+^ MBCs shared positive correlations with disease activity and severity, as well as the same side in PC1, indicating similar pathogenic abnormality. While naive B cells were negatively correlated with disease activity and severity, which seemed to be the anti-pathogenic factor opposite to plasmablasts in some aspects (3, 18). CD19^+^IgD^+^CD27^+^ unswitched MBCs were negatively correlated with T-IgE, but CD19^+^IgD^-^CD27^+^ MBCs were positively correlated. Given that unswitched MBCs are anti-inflammatory (26), we surmise T-IgE may play the opposite role in IgG4-RD, its increase may due to the unbalance between CD19^+^IgD^+^CD27^+^ unswitched MBCs and CD19^+^IgD^-^CD27^+^ MBCs. CD19^+^CD24^hi^CD38^hi^ Breg was negatively correlated with ESR, consisting with its function of IL-10 production (31), which is known as the anti-inflammatory cytokine. Our study also first proposed that the B-cell immunophenotype of aIgG4-RD patients consisted of plasmablast-naive B cell and MBCs-Breg axes abnormalities.

Based on B cell heterogeneity, aIgG4-RD patients were clearly classified into 3 subgroups. Subgroup1: lower MBCs-near normal Breg and naive B cells proportions; subgroup2: the highest MBCs-lowest Breg proportions; subgroup3: the highest plasmablasts cells-the lowest naive B cells proportions, as well as the highest disease activity and severity. Interestingly, we found that patients in subgroup2&3 seemed to be potential treatment-resistant: (i) patients in subgroup3 received the highest doses of initial GCs and stronger potency IM, but still had the highest serological biomarker levels even if disease achieved remission; (ii) although patients in subgroup1 and subgroup2 had similar levels of serological biomarkers at baseline, subgroup2 had no significant improvement in these biomarkers after treatments. The results further indicates that huge icebergs (e.g., B-cell heterogeneity, other uncharted territories) are below the tip of the iceberg (e.g., clinical manifestations, imaging examinations, IgG4-RD RI) in IgG4-RD, which help physicians in aiming at a complete assessment of individual patients for therapeutic decision-making, relapse risk prediction, and prognosis evaluation. Therapies targeting the B-cell lineage have been applied, including RTX targeting CD20 and XmAb5871 targeting CD19 (32). Our findings provide theoretical support for B-cell depletion applying in IgG4-RD, especially in refractory cases. Considering the B-cell heterogeneity and the imperfections of B-cell depletion, such as non-selective depletion, high rate of infections, the temporary effect (11), and its inability to prevent re-emergence of pathogenic plasmablasts (33), more precision medicine based on IgG4-RD heterogeneity is expected to perfect IgG4-RD treatment.

The association of B-cell subsets abnormalities and clinical phenotypes provided support for our findings that IgG4-RD was a heterogeneous condition immunologically with plasmablast-naive B cell and memory B cell-Breg axes abnormalities, patients with higher plasmablasts and MBCs had both higher serological biomarker levels and more serious clinical condition including more potential to be systemic involved and refractory. Conversely, relatively normal naïve and Breg, or lower CD19+IgD-CD27+ MBCs may be a mild sign to patients with IgG4-RD.

Our study has limitations. First, the sample size was limited. Therefore we cannot perform stratified analysis according to different IgG4-RD types, such as types of organs involved, and different treatments. We also didn’t observe clear relapse in subgroup2&3, although they had higher levels of risk factors after remission. Second, no patient received RTX in our study, we cannot comment on the impact of B-cell depletion on the B-cell immune signatures. Third, although we explored 10 B-cell immune subsets, B-cell set is complicated and continually updated. Further molecular and genetic characterization of B-cell subsets could have offered a better understanding of their involvement in the pathogenesis of IgG4-RD and will be the focus of future studies.

In conclusion, IgG4-RD is a heterogeneous condition immunologically with plasmablast-naive B cell and memory B cell-Breg axes abnormalities. Classification of patients with IgG4-RD based on B-cell immunophenotypes could help to identify potential refractory patients. A deeper understanding of these findings will improve our understanding of IgG4-RD pathogenesis, and lead to the development of more precise and effective therapies.

## Data Availability Statement

The original contributions presented in the study are included in the article/**Supplementary Material**. Further inquiries can be directed to the corresponding authors.

## Ethics Statement

The studies involving human participants were reviewed and approved by the medical ethics committee of Peking Union Medical College Hospital (Beijing, China). The patients/participants provided their written informed consent to participate in this study.

## Author Contributions

Design of research study: WZ and JL. Acquiring data: ZL, PZ, and WL. Data analysis: JL. Recruiting patients: HL, YP, LP, JZ, MW, HC, LZ, LW, CQ, and CH. Analyzing data: WZ and JL. Writing the manuscript: JL. Review of the manuscript: XZ, YZ, YF, and WZ. All authors contributed to the article and approved the submitted version.

## Funding

This work was supported by the National Natural Science Foundation of China (81771757, 81771780, 82071839), the Non-profit Central Research Institute Fund of Chinese Academy of Medical Sciences (NWB20203346), Capital’s Funds for Health Improvement and Research (No. 2020-2-4017) and Beijing Municipal Science & Technology Commission (No. Z201100005520023).

## Conflict of Interest

The authors declare that the research was conducted in the absence of any commercial or financial relationships that could be construed as a potential conflict of interest.

## Publisher’s Note

All claims expressed in this article are solely those of the authors and do not necessarily represent those of their affiliated organizations, or those of the publisher, the editors and the reviewers. Any product that may be evaluated in this article, or claim that may be made by its manufacturer, is not guaranteed or endorsed by the publisher.

## Supplementary Material

Supplementary Figure 1 Flow cytometry analysis of B cell subsets. **(A)** Gating strategy of B-cell subsets. **(B)** High percentage of plasmablasts and memory B cells in active IgG4-RD. SMB, CD19+IgD-CD27+ switched memory B cell, UMB, unswitched memory B cell.

Supplementary Figure 2 Results of hierarchical statistical cluster analysis based on immune cells in 105 IgG4-RD patients.

Supplementary Figure 3 Results of scatter gram based on B cell subsets in 105 patients with IgG4-RD. **(A)** The three-dimensional diagrams based on CD19+CD24-CD38hi plasmablast, CD19+CD24hiCD38hi Breg, and CD19+CD24+CD38- memory B cell. **(B)** The three-dimensional diagrams based on CD19+CD24intCD38int naive B cell, CD19+CD24+CD38- memory B cell, and CD19+CD24hiCD38hi Breg.

Supplementary Figure 4 Results of statistical cluster analysis based on 10 B-cell subsets in the mixed cohort.

Supplementary Figure 5 Principal components selected based on B-cell subsets in patients with active IgG4-RD. **(A)** Scree plot for cluster analysis. **(B)** Principal component loading of two axes. Blue for negative and red for positive. PC1: principal component 1, PC2: principal component 2.

Supplementary Figure 6 Distribution of clinical phenotypes in each group. **(A)** Distribution of clinical phenotypes compared between Group1 and Group2 identified by 4 B-cell subsets. **(B)** Distribution of clinical phenotypes compared among subgroups identified by 10 B-cell subsets. Phenotype, Clinical phenotypes of IgG4-related disease.

Supplementary Figure 7 Differences in B cell subsets among patients with different IgG4-RD clinical phenotypes. **(A)** Four B-cell subsets compared among patients with different clinical phenotypes in initial cohort of active IgG4-RD. n=105, one patient met none of the 4 clinical phenotypes. Total B, CD19+ B cells; Plasmablasts, CD19+CD24-CD38hi; Naïve B, CD19+CD24intCD38int; Memory B cell, CD19+CD24+CD38-; Regulatory B cell, CD19+CD24hiCD38hi. **(B)** Ten B-cell subsets compared among patients with different clinical phenotypes in the internal cohort of active IgG4-RD. n=49, four patient met none of the 4 clinical phenotypes. Phenotype, Clinical phenotypes of IgG4-related disease. Values are the mean ± SD, with levels compared with the healthy control group highlighted in color (blue for decreased; red for increased).
