# Supplementary figures and images for: Peripheral B-Cell Immunophenotyping Identifies Heterogeneity in IgG4-Related Disease
Source: Front Immunol. 2021 Sep 17;12:747076. doi: 10.3389/fimmu.2021.747076 (PMC8484311; doi:10.3389/fimmu.2021.747076)

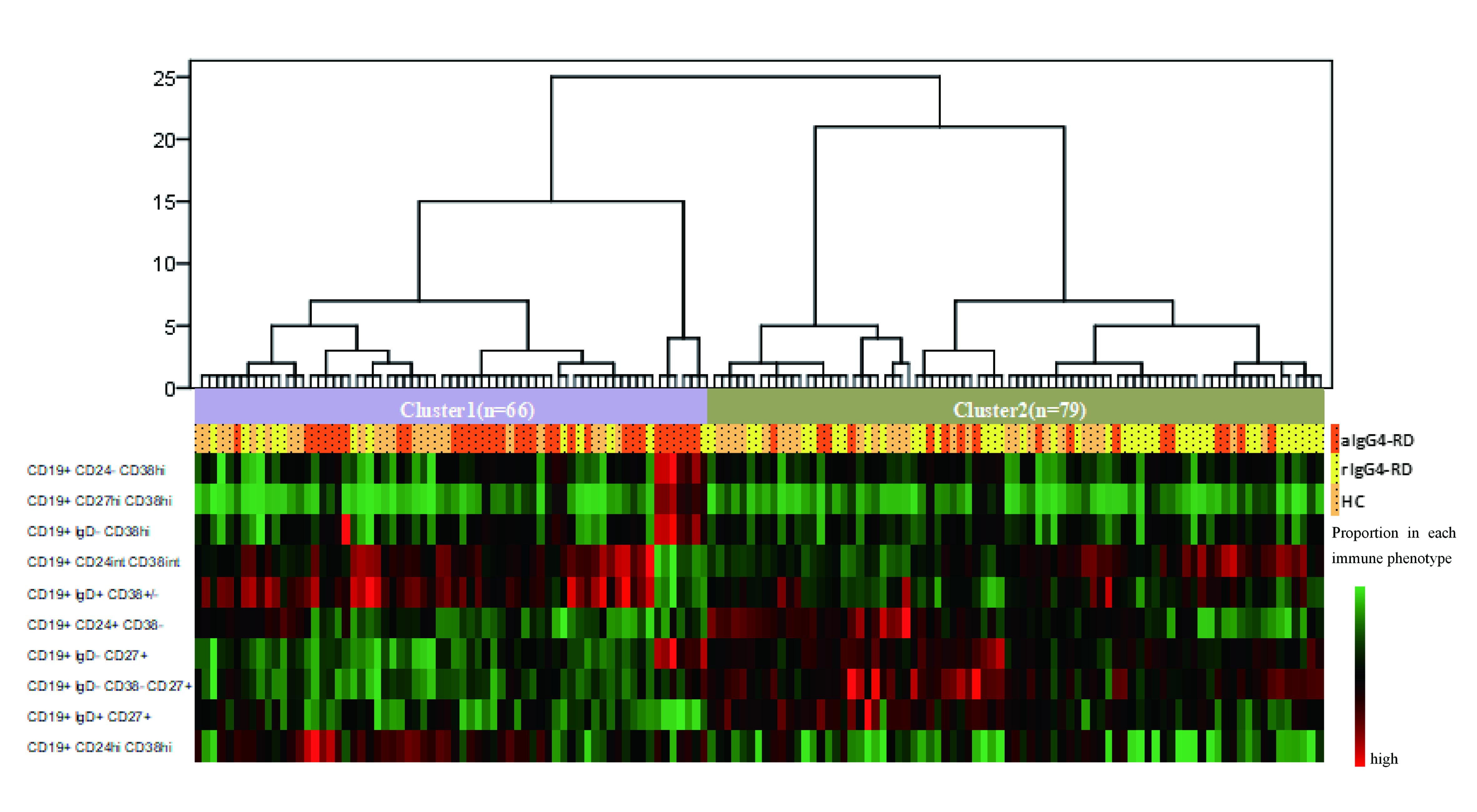

Supplement: Supplementary Figure 1 — Flow cytometry analysis of B cell subsets. (A) Gating strategy of B-cell subsets. (B) High percentage of plasmablasts and memory B cells in active IgG4-RD. SMB, CD19+IgD-CD27+ switched memory B cell, UMB, unswitched memory B cell. [file DataSheet_1.zip › Supplementary Figures/SF-4.tif]

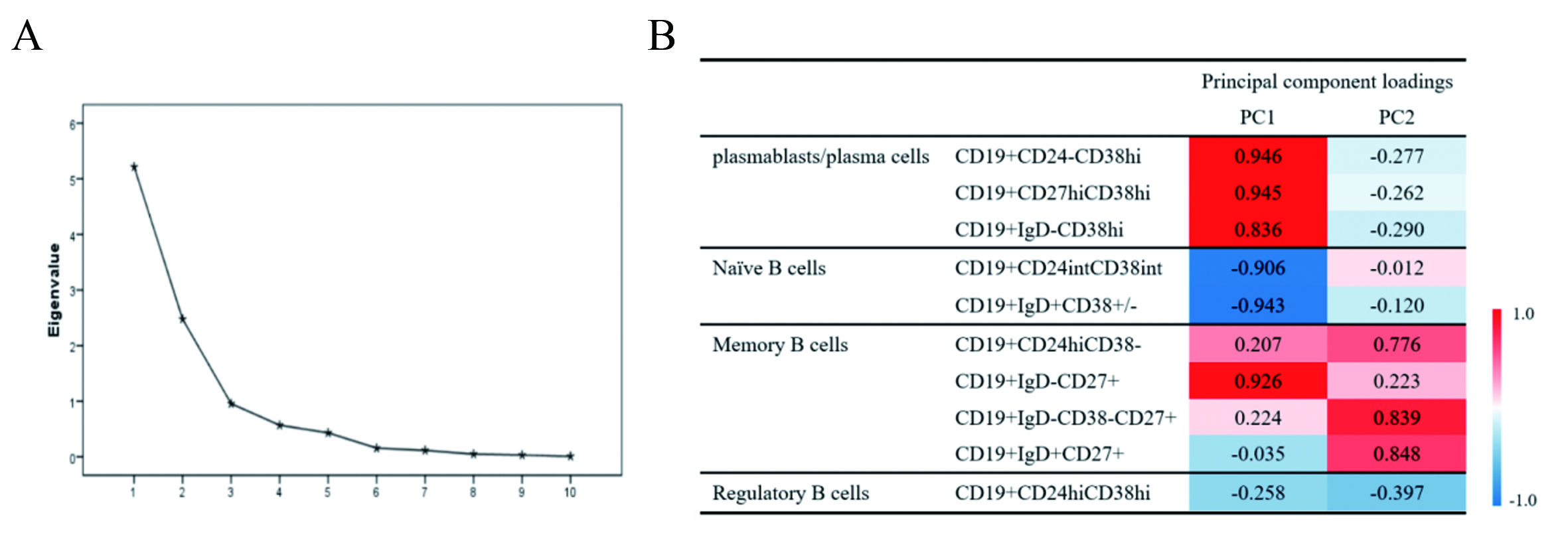

Supplement: Supplementary Figure 1 — Flow cytometry analysis of B cell subsets. (A) Gating strategy of B-cell subsets. (B) High percentage of plasmablasts and memory B cells in active IgG4-RD. SMB, CD19+IgD-CD27+ switched memory B cell, UMB, unswitched memory B cell. [file DataSheet_1.zip › Supplementary Figures/SF-5.tif]

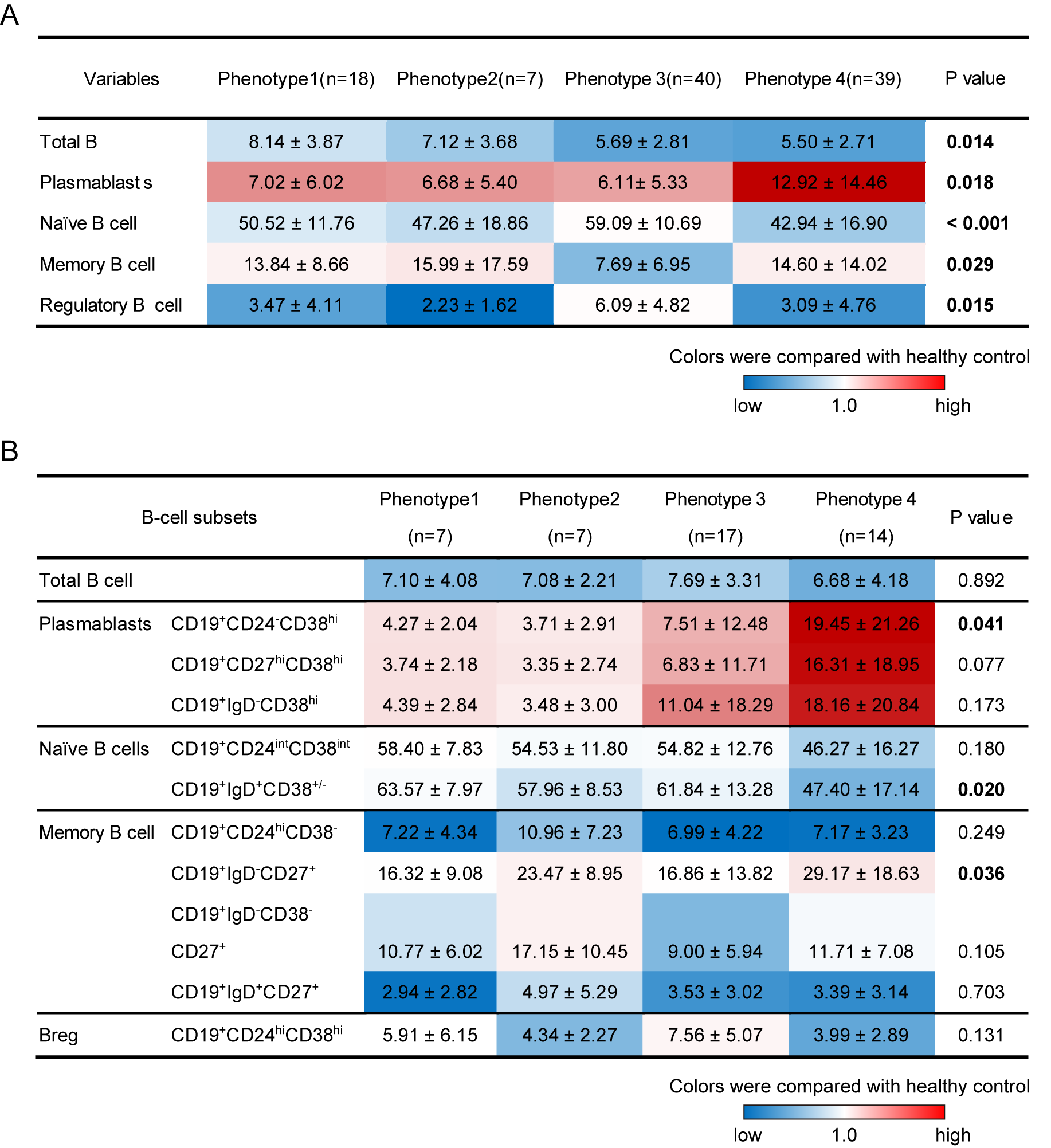

Supplement: Supplementary Figure 1 — Flow cytometry analysis of B cell subsets. (A) Gating strategy of B-cell subsets. (B) High percentage of plasmablasts and memory B cells in active IgG4-RD. SMB, CD19+IgD-CD27+ switched memory B cell, UMB, unswitched memory B cell. [file DataSheet_1.zip › Supplementary Figures/SF-7.tif]

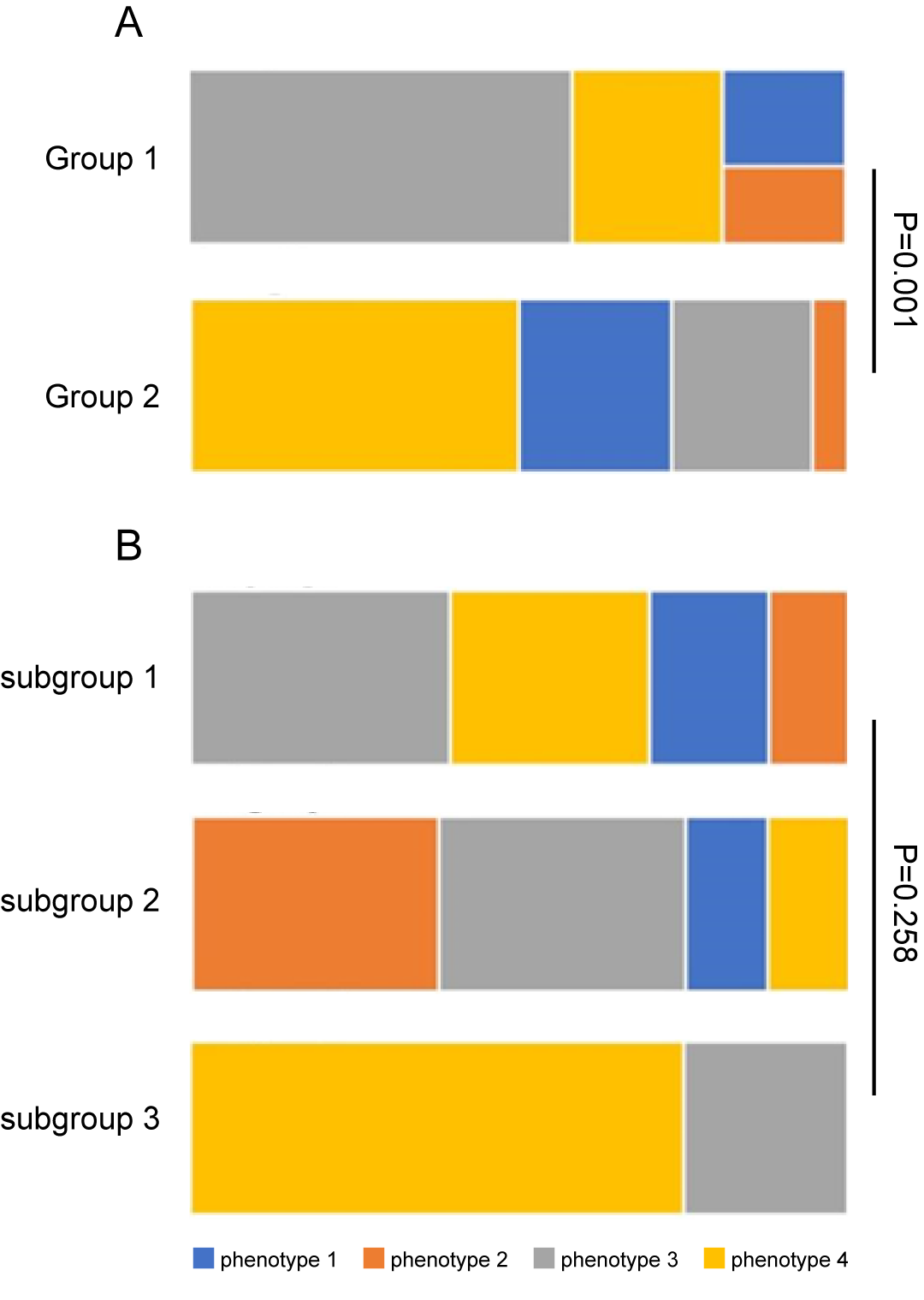

Supplement: Supplementary Figure 1 — Flow cytometry analysis of B cell subsets. (A) Gating strategy of B-cell subsets. (B) High percentage of plasmablasts and memory B cells in active IgG4-RD. SMB, CD19+IgD-CD27+ switched memory B cell, UMB, unswitched memory B cell. [file DataSheet_1.zip › Supplementary Figures/SF-6.tif]

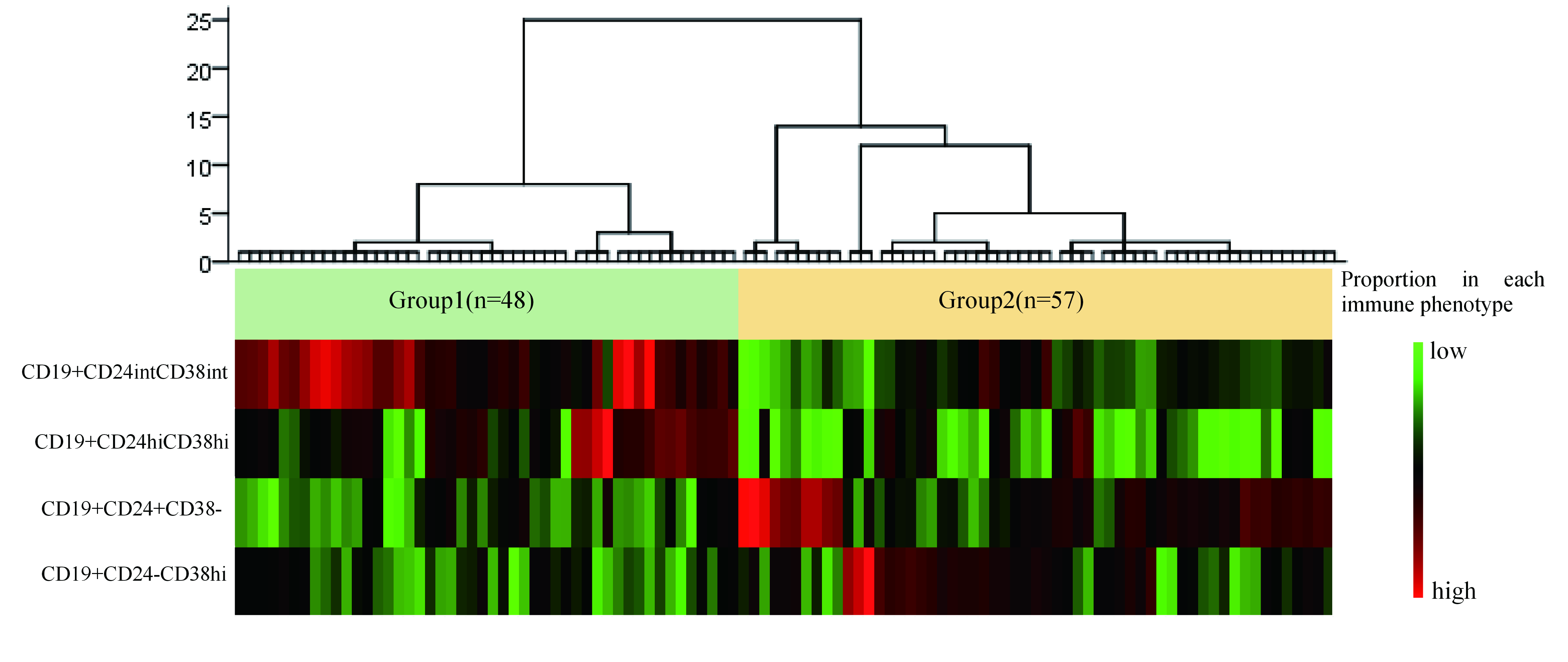

Supplement: Supplementary Figure 1 — Flow cytometry analysis of B cell subsets. (A) Gating strategy of B-cell subsets. (B) High percentage of plasmablasts and memory B cells in active IgG4-RD. SMB, CD19+IgD-CD27+ switched memory B cell, UMB, unswitched memory B cell. [file DataSheet_1.zip › Supplementary Figures/SF-2.tif]

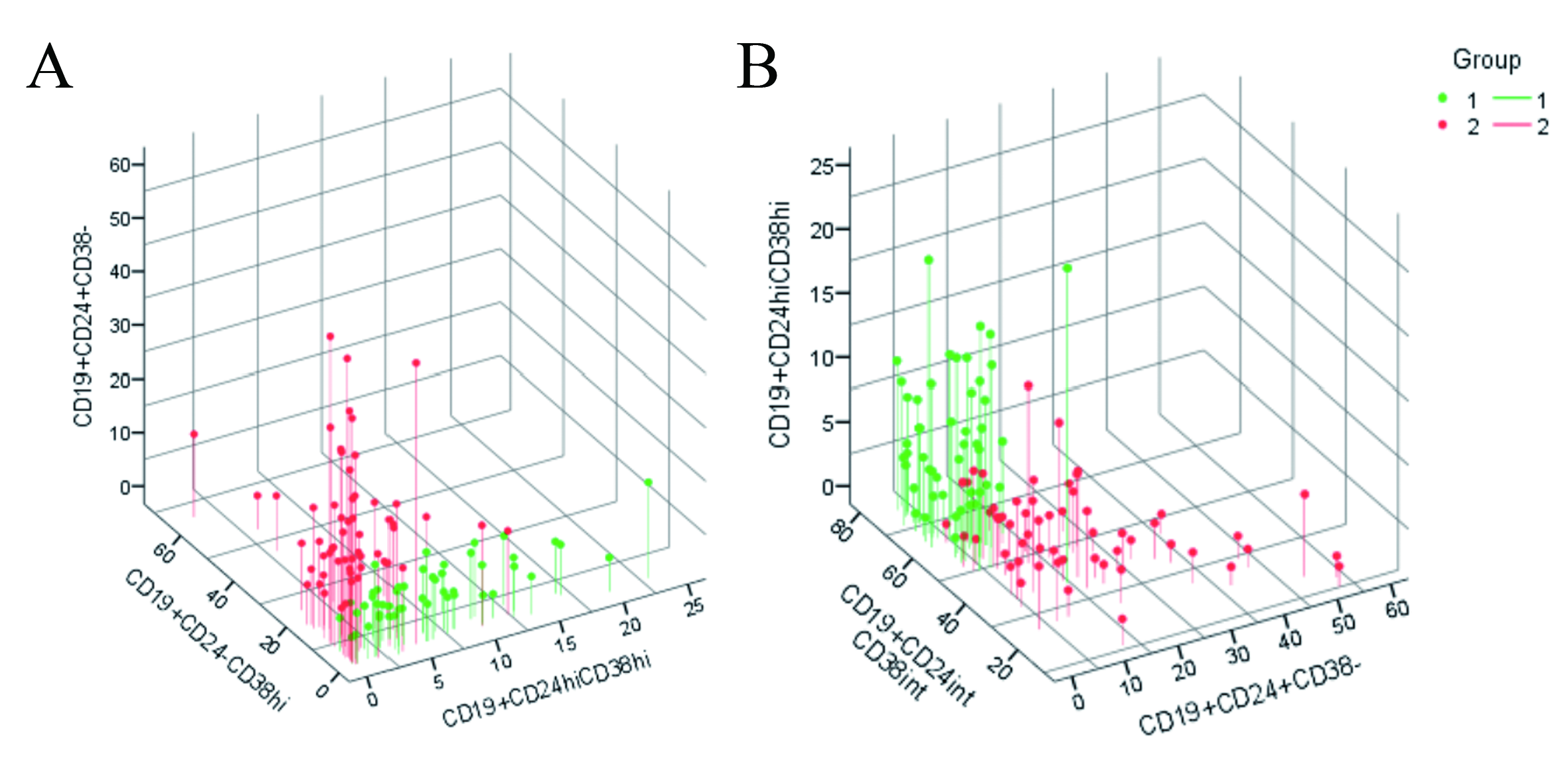

Supplement: Supplementary Figure 1 — Flow cytometry analysis of B cell subsets. (A) Gating strategy of B-cell subsets. (B) High percentage of plasmablasts and memory B cells in active IgG4-RD. SMB, CD19+IgD-CD27+ switched memory B cell, UMB, unswitched memory B cell. [file DataSheet_1.zip › Supplementary Figures/SF-3.tif]

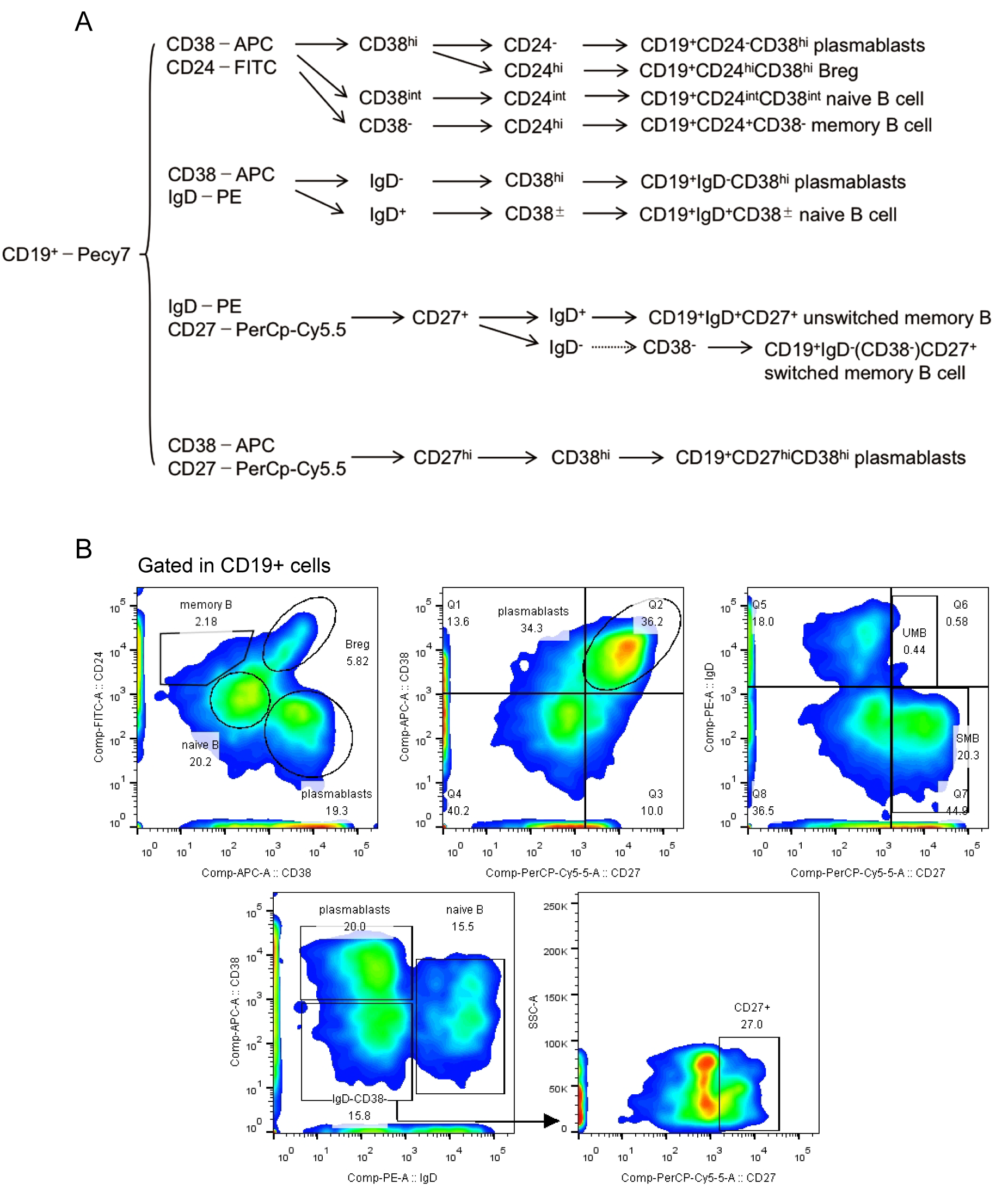

Supplement: Supplementary Figure 1 — Flow cytometry analysis of B cell subsets. (A) Gating strategy of B-cell subsets. (B) High percentage of plasmablasts and memory B cells in active IgG4-RD. SMB, CD19+IgD-CD27+ switched memory B cell, UMB, unswitched memory B cell. [file DataSheet_1.zip › Supplementary Figures/SF-1.tif]
