# Supplementary material for: Peripheral B-Cell Immunophenotyping Identifies Heterogeneity in IgG4-Related Disease
Source: Front Immunol. 2021 Sep 17;12:747076. doi: 10.3389/fimmu.2021.747076 (PMC8484311; doi:10.3389/fimmu.2021.747076)
Supplement: Supplementary file 2 [file Table_1.docx]

**Supplementary tables**

**Supplementary Table 1. Baseline characteristics and B cell subsets of the 105 IgG4-RD patients grouped according to cluster analysis.**

| Variables | Total(n=105) | Group1(n=48) | Group2(n=57) | P value | |
| --- | --- | --- | --- | --- | --- |
|  |  |  |  | Univariate | Multivariate |
| Age of onset (years) | 49.63 ± 12.90 | 48.22± 11.55 | 50.82 ± 13.92 | 0.311 |  |
| Age of diagnosis (years) | 52.50 ± 12.52 | 51.52 ± 10.64 | 53.31 ± 13.96 | 0.457 |  |
| Sex, n (% male) | 68( 64.8%) | 25 (52.1%) | 43 (75.4%) | 0.013 | 0.488 |
| Disease duration (months) | 36.88 ± 53.84 | 46.53 ± 66.55 | 28.79 ± 39.05 | 0.053 | **0.011** |
| Number of organs involved | 3.65 ±1.57 | 3.02 ± 1.40 | 4.25 ± 1.48 | < 0.001 | 0.242 |
| Diagnostic score* | 32.90 ± 10.60 | 30.4 ± 8.46 | 35.02 ± 11.79 | 0.047 | 0.494 |
| IgG (g/L) | 23.82 ± 11.68 | 18.88 ± 6.25 | 27.81 ±13.44 | < 0.001 | 0.694 |
| IgA (g/L) | 2.01 ± 1.19 | 2.13 ± 0.98 | 1.91 ± 1.34 | 0.131 |  |
| IgM (g/L) | 0.93 ± 0.75 | 0.89 ± 0.56 | 0.96 ± 0.88 | 0.631 |  |
| IgG1 (mg/L) | 10065.15 ±5386.50 | 8395.71 ± 2351.01 | 11295.26± 6564.89 | 0.008 | 0.653 |
| IgG2 (mg/L) | 5790.09 ± 3004.80 | 5499.52 ± 1832.24 | 6004.18 ± 3637.66 | 0.481 |  |
| IgG3 (mg/L) | 787.0 ± 751.05 | 591.60 ± 455.58 | 931.08 ± 886.23 | 0.065 |  |
| IgG4 (mg/L) | 18327.42±17117.26 | 11596.38±8701.34 | 23995.67± 20225.26 | 0.001 | 0.944 |
| IgE (KU/L) | 724.53 ± 991.34 | 576.97 ± 909.93 | 884.05 ± 1061.79 | 0.044 | 0.3 |
| 2018 RI | 7.60 ± 3.48 | 6.00 ± 2.86 | 8.95 ± 3.40 | < 0.001 | 0.59 |
| Total B | 6.23 ± 3.27 | 5.73 ± 2.99 | 6.65 ± 3.45 | 0.144 |  |
| Plasmablast | 8.79 ± 10.26 | 4.26 ± 2.34 | 12.59 ± 12.59 | < 0.001 | **0.006** |
| Naïve B cell | 50.71 ± 15.53 | 62.76 ± 8.44 | 40.55 ± 12.58 | < 0.001 | **0.001** |
| Memory B cell | 11.19 ± 11.46 | 5.39 ± 3.35 | 17.40 ± 12.93 | < 0.001 | **＜0.001** |
| Regulatory B cell | 4.24 ± 4,70 | 6.76 ± 3.32 | 2.12 ± 2.69 | < 0.001 | **0.001** |

P values in the univariate analysis were determined by one-way analysis of variance (ANOVA) or Kruskall-Wallis test. Multivariate analyses were performed using variables for which the P values in the univariate analysis were less than 0.05.

**Supplementary Table 2. Multiple comparisons among 3 cohorts.**

| **Variables** | |  | **versus** | **p value** |
| --- | --- | --- | --- | --- |
| sex | | aIgG4-RD | rIgG4-RD | 0.682 |
|  |  |  | HC | 1.000 |
|  |  | rIgG4-RD | HC | 0.541 |
| lymphocyte | | aIgG4-RD | rIgG4-RD | 0.573 |
|  |  |  | HC | 0.615 |
|  |  | rIgG4-RD | HC | 0.956 |
| Total B cell | | aIgG4-RD | rIgG4-RD | 0.127 |
|  |  |  | HC | 0.047 |
|  |  | rIgG4-RD | HC | 0.006 |
| plasmablasts | CD19+CD24-CD38hi | aIgG4-RD | rIgG4-RD | ＜0.001 |
|  |  |  | HC | ＜0.001 |
|  |  | rIgG4-RD | HC | 0.075 |
|  | CD19+CD27hiCD38hi | aIgG4-RD | rIgG4-RD | ＜0.001 |
|  |  |  | HC | ＜0.001 |
|  |  | rIgG4-RD | HC | 0.286 |
|  | CD19+IgD-CD38hi | aIgG4-RD | rIgG4-RD | ＜0.001 |
|  |  |  | HC | ＜0.001 |
|  |  | rIgG4-RD | HC | 0.717 |
| Naïve B cells | CD19+CD24intCD38int | aIgG4-RD | rIgG4-RD | 0.629 |
|  |  |  | HC | 0.358 |
|  |  | rIgG4-RD | HC | 0.659 |
|  | CD19+IgD+CD38+/- | aIgG4-RD | rIgG4-RD | 0.042 |
|  |  |  | HC | 0.004 |
|  |  | rIgG4-RD | HC | ＜0.001 |
| Memory B cells | CD19+IgD-CD27+ | aIgG4-RD | rIgG4-RD | 0.577 |
|  |  |  | HC | 0.05 |
|  |  | rIgG4-RD | HC | 0.012 |
|  | CD19+IgD-CD38-CD27+ | aIgG4-RD | rIgG4-RD | 0.656 |
|  |  |  | HC | 0.772 |
|  |  | rIgG4-RD | HC | 0.880 |
|  | CD19+IgD+CD27+ | aIgG4-RD | rIgG4-RD | 0.066 |
|  |  |  | HC | 0.023 |
|  |  | rIgG4-RD | HC | 0.641 |
| Regulatory B cells | CD19+CD24hiCD38hi | aIgG4-RD | rIgG4-RD | ＜0.001 |
|  |  |  | HC | 0.261 |
|  |  | rIgG4-RD | HC | ＜0.001 |

**Supplementary Table 3. Correlations between disease activity and peripheral cell phenotype at baseline by univariate analysis.**

| Gated in CD19+ |  | organs | RI | IgG | IgG1 | IgG2 | IgG3 | IgG4 | T-IgE | EOS% | AEC | ESR | C3 |
| --- | --- | --- | --- | --- | --- | --- | --- | --- | --- | --- | --- | --- | --- |
| CD24-CD38hi | r | **.311*** | **.310*** | **.715**** | **.499**** | **.326*** | **.466**** | **.593**** | **.550**** | **.629**** | **.601**** | **.499**** | **-.464**** |
|  | p | 0.033 | 0.032 | 0.000 | 0.000 | 0.027 | 0.001 | 0.000 | 0.000 | 0.000 | 0.000 | 0.000 | 0.003 |
| CD27hiCD38hi | r | 0.272 | 0.271 | **.724**** | **.514**** | **.349*** | **.501**** | **.592**** | **.575**** | **.668**** | **.642**** | **.487**** | **-.482**** |
|  | p | 0.064 | 0.063 | 0.000 | 0.000 | 0.017 | 0.000 | 0.000 | 0.000 | 0.000 | 0.000 | 0.001 | 0.002 |
| IgD-CD38hi | r | **.324*** | **.320*** | **.552**** | **.385**** | 0.276 | **.392**** | **.493**** | **.465**** | **.538**** | **.502**** | **.376*** | **-.368*** |
|  | p | 0.026 | 0.027 | 0.000 | 0.008 | 0.063 | 0.007 | 0.000 | 0.001 | 0.000 | 0.001 | 0.011 | 0.023 |
| CD24intCD38int | r | **-.295*** | -0.280 | **-.644**** | **-.511**** | **-.311*** | **-.413**** | **-.455**** | **-.427**** | **-.515**** | **-.515**** | **-.410**** | **.526**** |
|  | p | 0.044 | 0.054 | 0.000 | 0.000 | 0.035 | 0.004 | 0.001 | 0.003 | 0.000 | 0.000 | 0.005 | 0.001 |
| IgD+CD38+/- | r | **-.311*** | **-.327*** | **-.609**** | **-.502**** | **-.294*** | **-.472**** | **-.441**** | **-.442**** | **-.550**** | **-.553**** | **-.458**** | **.470**** |
|  | p | 0.034 | 0.023 | 0.000 | 0.000 | 0.048 | 0.001 | 0.002 | 0.002 | 0.000 | 0.000 | 0.002 | 0.003 |
| IgD-CD27+ | r | 0.206 | 0.194 | **.624**** | **.518**** | **.387**** | **.506**** | **.351*** | **.444**** | **.535**** | **.537**** | **.448**** | **-.506**** |
|  | p | 0.165 | 0.186 | 0.000 | 0.000 | 0.008 | 0.000 | 0.014 | 0.002 | 0.000 | 0.000 | 0.002 | 0.001 |
| IgD-CD38-  CD27+ | r | 0.016 | 0.026 | -0.009 | 0.183 | 0.220 | 0.122 | -0.266 | -0.114 | -0.098 | -0.035 | 0.141 | -0.196 |
|  | p | 0.913 | 0.858 | 0.955 | 0.225 | 0.143 | 0.420 | 0.067 | 0.455 | 0.526 | 0.821 | 0.354 | 0.238 |
| IgD+CD27+ | r | -0.014 | -0.029 | -0.235 | -0.164 | -0.124 | -0.234 | -0.277 | **-.349*** | -0.215 | -0.184 | -0.240 | -0.025 |
|  | p | 0.924 | 0.847 | 0.112 | 0.277 | 0.410 | 0.117 | 0.057 | 0.019 | 0.161 | 0.233 | 0.112 | 0.883 |
| CD24hiCD38hi | r | -0.079 | -0.105 | -0.066 | -0.059 | -0.019 | -0.168 | -0.093 | -0.138 | -0.080 | -0.034 | **-.303*** | 0.103 |
|  | p | 0.597 | 0.478 | 0.661 | 0.696 | 0.899 | 0.265 | 0.529 | 0.367 | 0.606 | 0.825 | 0.043 | 0.540 |

Organs, the number of organs involved; RI, IgG4-RD RI (2018); EOS%, percentage of eosinophil; AEC, absolute eosinophil count; ESR, erythrocyte sedimentation rate; C3, complement 3, Ig, immunoglobulin; T-IgE, total-IgE. **. P＜0.01；*. P＜0.05. r, correlation coefficient, blue for negative and red for positive correlation.

**Supplementary Table 4. Multiple comparisons of patients’ serological biomarker levels based on cluster analysis among subgroups.**

| Variables |  | versus | p value |
| --- | --- | --- | --- |
| IgG | subgroup1 | subgroup2 | 0.999 |
|  |  | subgroup3 | ＜0.001 |
|  | subgroup2 | subgroup3 | 0.001 |
| IgG4 | subgroup1 | subgroup2 | 0.141 |
|  |  | subgroup3 | 0.001 |
|  | subgroup2 | subgroup3 | ＜0.001 |
| T-IgE | subgroup1 | subgroup2 | 0.129 |
|  |  | subgroup3 | 0.007 |
|  | subgroup2 | subgroup3 | 0.001 |
| ESR | subgroup1 | subgroup2 | 0.808 |
|  |  | subgroup3 | 0.006 |
|  | subgroup2 | subgroup3 | 0.010 |
| EOS% | subgroup1 | subgroup2 | 0.229 |
|  |  | subgroup3 | 0.001 |
|  | subgroup2 | subgroup3 | ＜0.001 |
| C3 | subgroup1 | subgroup2 | 0.821 |
|  |  | subgroup3 | 0.028 |
|  | subgroup2 | subgroup3 | 0.081 |
